# A cohort study of infant feeding practices in city, suburban and rural areas in Zhejiang Province, PR China

**DOI:** 10.1186/1746-4358-3-4

**Published:** 2008-03-03

**Authors:** Liqian Qiu, Yun Zhao, Colin W Binns, Andy H Lee, Xing Xie

**Affiliations:** 1 Women's Hospital, School of Medicine, Zhejiang University, PR China; 2 School of Public Health, Curtin University, WA, Australia

## Abstract

**Background:**

Breastfeeding is the basis for appropriate nutrition for infants and is strongly supported by the Ministry of Health in China. However, there are differences in infant feeding practices in different areas of the country. The aim of this study was to compare the infant feeding practices and the prevalence of determinants of initiation and continuing to breastfeed until six months of age in city, suburb and rural areas in Zhejiang Province, PRC.

**Methods:**

A longitudinal cohort study of infant feeding practices was undertaken in city, suburban and rural areas in 2004/2005. Mothers were recruited and interviewed before discharge from hospitals. A total of 1520 mothers were recruited into the study. Follow-up interviews were administered at 1, 3 and 6 months after birth to obtain details of infant feeding practices.

**Results:**

'Any breastfeeding' rates were high before discharge at all three locations, 96.5% in city, 96.8% in suburb and 97.4% in the rural area. The 'exclusive breastfeeding' rates in the city, suburban and rural areas before discharge were 38.0%, 63.4% and 61.0% respectively. By sixth months the 'any breastfeeding' rates had declined to 62.8%, 76.9% and 83.6% and the 'exclusive breastfeeding' rates had fallen to 0.2%, 0.5% and 7.2% in city, suburb and rural areas respectively. There were differences in feeding practices between the three locations, including the use of prelacteal feeds and the introduction of supplementary feeds.

**Conclusion:**

Mothers who lived in the city were least likely to be 'exclusive breastfeeding' at discharge. At six months the city infants also had lower rates of 'any breastfeeding' and 'exclusive breastfeeding'.

## Background

The Ministry of Health in China has recognized the importance of breastfeeding in infant nutrition and recommends exclusive breastfeeding for the first six months of life [1]. Like other developing countries, China was influenced in the 19^th ^and 20^th ^centuries by western practices and its traditional pattern of home-based delivery became hospital based. Mothers were often separated from their infants in hospitals and infant formula became more readily available. These factors and the improving economy led to a decline in the breastfeeding rate. In Shanghai, one of China's largest cities, a large cross-sectional study was conducted in 1980, supported by the World Health Organization (WHO) which included a total of 3845 mothers recruited from the city and suburbs [2]. The data from this study showed the 'any breastfeeding' rate had declined to 24.8% in the city and 77.0% in the suburbs for 0–6 month old babies [2]. This study was technically supported by WHO and used the period prevalence method of recording breastfeeding rates recommended by WHO [3]. In the following years further surveys revealed similar trends in other regions of the country. In 1983 a national cross-sectional survey of 111,348 infants aged 0–6 months found that the 'any breastfeeding' rate was 49.3% in the city and 75.1% in rural areas [4]. The decline in breastfeeding rates was a challenge for China as she sought to achieve the goals set at the International Child Survival Conference in 1990 which were endorsed by the Chinese Premier [1].

The Chinese government strongly supported the international goals as a way of improving the nutritional status of her children and in the early 1990s the Ministry of Public Health in China began promoting breastfeeding on a large scale across the nation. Several projects were launched to encourage breastfeeding and promote its benefits to parents and health professionals. These projects included initiating scientific research on the promotion of breastfeeding, promoting 'rooming in' and increasing health education about the benefits of breastfeeding. The State Council passed regulations to extend maternity leave from six weeks to three months to support breastfeeding. Employers were instructed to ensure that mothers had sufficient time for nursing if continuing to breastfeed and nursing rooms were required to be provided in work units. At the same time the first draft of the "National Program for the Promotion of Breast Feeding in China" was circulated [1]. The promotion of the "Baby Friendly Hospital Initiative" was commenced in China and initially a few maternity hospitals were accredited, followed by some of the larger integrated hospitals. Breastfeeding rates began to rise in the 1990s as "Baby Friendly accreditation" promoted by the Ministry of Health, spread across the country.

**Figure 1:**
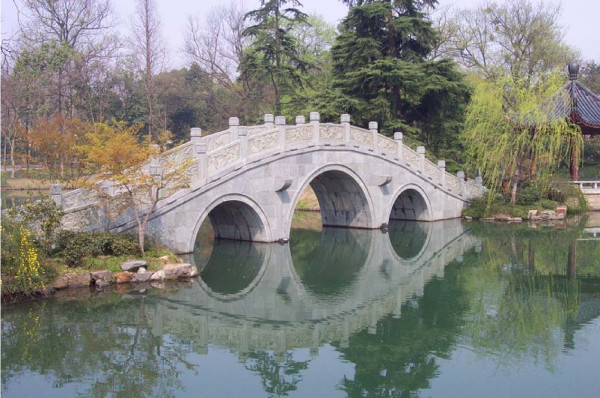
West Lake, Hangzhou.

Zhejiang Province is located on the east coast of China, south of Shanghai and in 2006 had a population of 49 million. Since the Chinese economic reforms began 30 years ago, Zhejiang Province has developed its economy, education and health care systems and has become one of the most prosperous regions in the nation. The capital of Zhejiang Province is Hangzhou, a city of four million, first made famous when Marco Polo was appointed its governor in the 12^th ^century. Hangzhou has become the centre of large information technology and electronics industries (Figure 1). Like other large cities in China, in Hangzhou, the breastfeeding rate declined with the introduction of western patterns of obstetric services. However, after the nationwide launch of the Baby Friendly Hospital Initiative in China, more than 85% of maternity and integrated hospitals in Zhejiang Province changed their existing systems to become "Baby Friendly" by meeting the WHO/UNICEF criteria. However, some barriers to breastfeeding still exist in hospitals and in the community and exclusive breastfeeding of infants up to six months of age is uncommon in Zhejiang Province. The rate is much lower than Chinese and international targets. This has important implications for the health of children of the province.

Because breastfeeding statistics are incomplete and there is little information about the reasons for breastfeeding and 'not breastfeeding', a longitudinal cohort study was commenced in Zhejiang Province. The aim of the study was to determine the prevalence of breastfeeding and prevalence of determinants of initiation and continuing to breastfeed until six months of age in city, suburban and rural areas of Zhejiang Province.

## Methods

A longitudinal cohort study of infant feeding practices was undertaken in three locations in Zhejiang Province, in the capital city (Hangzhou), in a suburban location (Fuyang) which is located 50 km to the southwest of Hangzhou and in a mountainous rural area a further 300 km to the south-west. All the mothers in the study were recruited while in hospital during the period October 2004 to December 2005. The first interview was undertaken by a nurse or women's health worker before discharge from hospital and follow up interviews were held at one month, three months and six months postpartum. The first interview was always undertaken in person and most of the follow-up interviews (92%) were undertaken by telephone. In the few instances where mothers could not be reached by telephone, the follow-up interviews were completed at the routine examinations in the community child care clinics. A total of 1520 mothers were recruited from four hospitals; the Women's Hospital, School of Medicine, Zhejiang University in the city, Fu-yang Maternal and Child Hospital in the suburban location and Jin-Yun People's Hospital and Li-Shui Maternal and Child Hospital in the rural area. Each of these hospitals is typical of the health care facilities in the area they are located. The inclusion criteria for the study were that the mother had delivered a live child, the mother and neonate did not have serious diseases and that she was resident in the service area of that hospital. In Hangzhou almost one half of the births were not of local residents and were excluded from the study. However, in the suburban and rural areas almost all mothers were local residents and were eligible to participate. In order to be able to manage the number being interviewed on any one day, selection was made using a series of random numbers. The response rate was high and 98.0% of mothers (1520 out of 1551) agreed to participate in the study.

The questionnaires were based on those developed by Scott, Binns, Xu and Duong that have been used extensively in breastfeeding cohort studies in Australia, Xinjiang, China and Vietnam [5-7]. The questionnaires were designed to identify the feeding method and to collect information on factors associated with breastfeeding. After translation the questionnaires were pilot tested in city and rural areas and were modified for the Zhejiang language and culture.

The project was approved by the Research Administration Section of the Women's Hospital, School of Medicine, Zhejiang University and the Human Research Ethics Committee of Curtin University, Australia. The purpose of the study was explained to the mothers and assurance was given that all information would be kept confidential. Any mother had the right to withdraw from the study at anytime without prejudice. After the purpose of the study had been explained to the mothers, they were given a consent letter to sign. The questionnaire and the interviewing nurses used standard terminology and the local dialect to ensure mothers' understanding.

All data analyses were carried out using the Statistical Package for the Social Sciences, release 14.0 (SPSS Inc.). Descriptive statistics and cross-tabulations were generated for demographic factors, life tables were used for breastfeeding rates and binary logistic regression was used to calculate odds ratio of infant formula use.

The definitions of breastfeeding used in this paper were based on standard definitions and were the same as those used in Xu's study in Xinjiang Province, in the west of China [6]:

• *Any breastfeeding*: The infant receives breastmilk (direct from the breast or expressed) with or without any other drink, formula or other infant food.

• *Exclusive breastfeeding*: Breastfeeding while giving no other food or liquid, not even water, with the exception of drops or syrups consisting of vitamins, mineral supplements or medicine.

Prelacteal feeds are defined as any feeds given before the onset of lactogenesis II, which is the onset of copious lactation that occurs within four days of birth [8].

## Results

A total of 1520 mothers were recruited into the study, 42.0% from the city, 22.8% from the suburban area and 35.2% from the rural area. Almost all mothers were married (99.9%), and most belonged to the Han ethnic group (97.5%). The details of the study sample and the prevalence of the major demographic variables in the city, suburban and rural areas are shown in Table 1.

**Table 1:** Demographic details of mothers' in the city, suburb and rural areas, Zhejiang Province, People's Republic of China, 2004–2005 (n = 1520)

**Variable**	**City**		**Suburb**		**Rural**		**Total**	
	**n**	**%**	**n**	**%**	**n**	**%**	**n**	**%**
**Residence**	638	42.0	347	22.8	535	35.2	1520	100.0

Maternal Age (years)*								
<25	77	12.0	124	35.7	158	30.6	358	23.9
25–29	383	60.2	165	47.6	255	49.2	800	53.5
30–34	151	23.9	51	14.7	80	15.5	282	18.9
≥35	25	3.9	7	2.0	24	4.7	56	3.7
Missing	5		0		19		24	
Maternal education (years)*								
≤9	57	9.0	206	59.4	282	53.1	544	36.0
10–12	172	26.9	78	22.5	121	22.9	370	24.5
>12	407	64.1	63	18.2	127	24.0	597	39.5
Missing	3				6		9	
Maternal employment*								
Labour job	67	10.5	188	55.1	238	47.1	492	33.3
Office job	494	78.4	90	26.4	179	35.4	763	51.7
Not employed	70	11.1	63	18.5	89	17.4	221	15.0
Missing	8		6		30		44	
Gestation (weeks)*								
<37	30	4.7	7	2.0	12	2.4	49	3.3
≥37	605	95.3	340	98.0	499	97.6	1443	96.7
Missing	3		0		25		28	
**Birth weight (g)**								
<2500	9	1.4	7	2.0	11	2.1	27	1.8
2500–3999	583	91.8	310	89.3	489	93.3	1382	91.8
≥4000	43	6.8	30	8.6	24	4.6	97	6.4
Missing	3		0		11		14	
Delivery method*								
Vaginal	155	24.3	90	25.9	252	47.2	495	32.7
Caesarean	481	75.7	257	74.1	281	52.8	1019	67.3
Missing	3		0		3		6	
Birth order*								
1	615	96.9	290	84.1	442	83.4	1347	89.2
≥2	20	3.1	55	15.9	89	16.6	163	10.8
Missing	3		2		5		10	
**Baby's gender**								
Male	347	54.2	162	47.4	260	49.7	767	51.1
Female	291	45.8	180	52.6	263	50.3	734	48.9
Missing	2		5		12		19	
Monthly family income* (RMB)								
≤1500	6	1.0	51	15.0	158	31.3	215	14.6
1501–3000	75	11.9	145	42.8	182	35.9	401	27.2
3001–5000	227	35.7	97	28.6	129	25.6	451	30.6
>5000	326	51.5	46	13.6	36	7.1	407	27.6
Missing	7		8		31		46	

* p < 0.05

Generally the mothers from the city had higher levels of education, a higher proportion of office employment and higher family income compared to the mothers in the suburban and rural areas. Mothers from the city usually were older and only 12.0% of mothers in the city had their babies before the age of 25 years compared to 35.7% in the suburban and 30.6% in the rural locations. Mothers in the city and suburban area were more likely to give birth by caesarean section: 75.7% in the city and 74.1% in the suburban area, compared to 52.8% in the rural area.

The proportions of mothers who were breastfeeding before hospital discharge from the three locations are shown in Table 2. More than 95% of mothers in each location initiated breastfeeding, but the exclusive breastfeeding rate was much lower in the city. A high proportion of mothers used prelacteal feeds on at least one occasion before discharge as previously reported [9].

**Table 2:** 'Any breastfeeding', 'exclusive breastfeeding' and prelacteal feeding rates at discharge in Zhejiang Province, PR China, 2004–2005

	**'Any breastfeeding'**		**'Exclusive breastfeeding'**		**Prelacteal feeding**	
	**n**	**%**	**n**	**%**	**n**	**%**
**City**	613	96.5	233	38.0	380	62.0
**Suburb**	336	96.8	213	63.4	123	36.6
**Rural**	518	97.4	316	61.0	202	39.0

**Figure 2:**
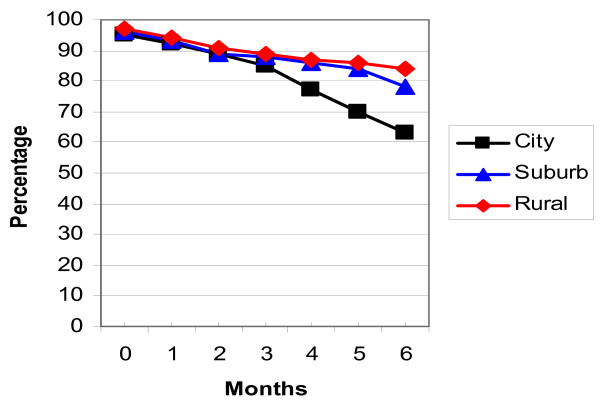
'Any breastfeeding' rates Zhejiang Province.

**Figure 3:**
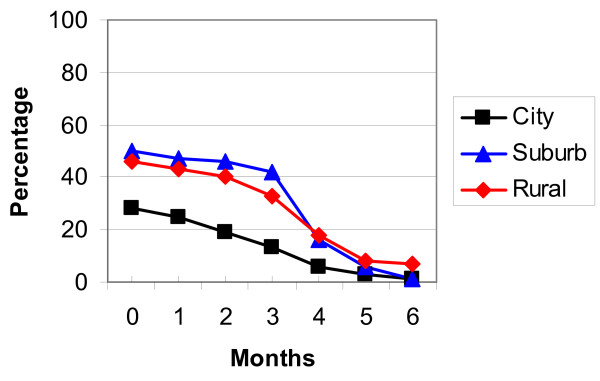
'Exclusive breastfeeding' rates Zhejiang Province.

The breastfeeding rates in the three locations were analysed using life table analysis and are detailed in Table 3, Figures 2 and 3. Figure 2 shows the 'any breastfeeding' rates and Figure 3 the 'exclusive breastfeeding' rates for the three locations in Zhejiang Province. While the majority of mothers were continuing to breastfeed at six months, only a few infants were exclusively breastfed at this age, especially in the city and suburbs.

**Table 3:** 'Any breastfeeding' and 'exclusive breastfeeding' rates from 1–6 months in city, suburban and rural Zhejiang Province, PR China

**Location**	**Age months**	**'Any breastfeeding'**			**'Exclusive breastfeeding'**		
		**n**	**%**	**95%CI**	**n**	**%**	**95%CI**
**City**	1~	590	91.7	89.4,93.9	166	24.4	21.0,27.7
	2~	380	88.6	85.8,91.3	119	18.8	15.6,22.0
	3~	367	84.0	80.7,87.2	92	13.7	10.8,16.6
	4~	347	76.2	72.3,80.1	67	5.5	3.5,7.5
	5~	315	69.7	65.4,74.0	27	1.8	0.7,3.0
	6~	288	62.8	57.7,67.9	9	0.2	NA
**Suburb**	1~	325	90.4	87.3,93.5	174	47.0	41.7,52.3
	2~	311	88.4	85.0,91.8	161	45.8	40.6,51.1
	3~	304	86.6	83.0,90.2	157	41.7	36.5,46.9
	4~	290	84.5	80.7,88.3	140	16.1	12.2,20.0
	5~	283	82.7	78.7,86.7	54	4.5	2.3,6.7
	6~	277	76.9	71.8,82.0	15	0.5	NA
**Rural **	1~	499	93.5	91.4,95.6	201	42.7	38.1,47.3
	2~	486	90.6	88.1,93.1	189	39.8	35.2,44.3
	3~	471	89.0	86.4,91.7	176	33.1	28.8,37.5
	4~	456	86.9	84.0,89.8	143	18.3	14.7,21.9
	5~	445	85.1	82.1,88.2	79	8.1	5.5,10.7
	6~	436	83.6	80.2,87.0	35	7.2	4.7,9.8

The exclusive breastfeeding rate was lower than the national target (80% till four months of age) in all locations and at all ages. A lower proportion of mothers in the city (38.0%) were exclusively breastfeeding compared to the suburban (63.4%) and rural (61.0%) areas on discharge.

The prevalence data were converted to period prevalence, to allow comparison with WHO statistics and are shown in Table 4.

**Table 4:** The period prevalence (0–6 months) of 'any' and 'exclusive' breastfeeding in Zhejiang Province, PR China

		**'Any breastfeeding'**	**'Exclusive breastfeeding'**
	**n**	**%**	**%**
**City**	406	81.1	13.0
**Suburb**	342	86.0	29.5
**Rural**	530	89.2	27.8

Supplementary feeding with infant formula and other complementary foods is quite common in China. The prevalence of formula feeding at the different locations at three and six months of age is described in Table 5.

**Table 5:** The prevalence of infant formula or other complementary food use in city, suburb and rural areas Zhejiang Province, PR China

	**Using infant formula or other complementary food at 3 months**				**Using infant formula or other complementary food at 6 months**			
	**n**	**%**	OR*	**95% CI**	**n**	**%**	OR*	**95% CI**
**Rural**	530	42.3	1		469	89.8	1	
**City**	406	32.7	2.10	1.61,2.73	705^#^	99.0	11.38	5.13,25.24
**Suburb**	342	60.6	0.67	0.50,0.88				

*OR = crude odds ratio.

^#^City and suburban data are combined to calculate the Odds Ratio at 6 months

In Table 5, the crude odds ratios and confidence intervals were calculated using binary logistic regression analysis. Compared with mothers living in rural areas, mothers who lived in the city were more likely to use formula by the third month after birth (OR: 2.10, 95%CI: 1.61, 2.73). Mothers who lived in the suburban area were less likely to be using formula feeding (OR: 0.67, 95%CI: 0.50, 0.88).

By the sixth month very few mothers in both city and suburban area were exclusively breastfeeding (0.2% and 0.5% respectively), but in the rural area the rate was higher where 7.2% of mothers were still exclusively breastfeeding. The infant feeding practices of the mothers from the city and suburban area were similar and when these two groups were combined and compared to the rural group the contrast at six months was even stronger (Crude OR:11.38, 95%CI: 5.13, 25.24).

## Discussion

The economy of Zhejiang Province has grown rapidly in recent years, particularly since the beginning of the 21^st ^century and the per capita GDP in Hangzhou was $6,700 in 2006. The rise in living standards accompanying the economic growth has led to a demand for improved health care and in particular, the application of advanced medical technologies. Infant feeding practices are another part of the culture which has been influenced by economic development. There is now widespread promotion of infant formula and mothers are fascinated by the prospect of a high-technology product which promises much for their infants. On the other hand, cultural beliefs are still strong and most mothers commence breastfeeding, but they tend to combine this with infant formula in the early months of their infants' lives.

Chinese society has changed rapidly and in the "high-tech" city of Hangzhou it is now very common for women to pursue higher education and to marry later in life. The women of Hangzhou are highly educated and in our study 64.1% of the women had post secondary education compared with the rates of 18.2% in the suburban area and 24.0% in the rural area. Almost half of the mothers (45.5%) considered for recruitment into the study were not native residents of the city, compared to the suburban (18.4%) and the rural areas (3.7%). This reflects the dynamic nature of the Hangzhou population resulting from the rapid economic development.

There have been rapid changes in medical practice in the past decades and this is reflected in changes in birthing methods. Overall in the study, two thirds of mothers gave birth by caesarean section. The rates in the city and suburban and rural areas were 75.7%, 74%, and 52.7% respectively. After the Baby Friendly Hospital Initiative (BFHI) was promoted in China in the 1990s, the majority of hospitals in the Province reformed their obstetric practices. The hospital environment became friendly towards babies, as the Ten Steps to Successful Breastfeeding were required in all hospitals and maternal and child health centres. These included 'rooming in', early skin contact, early initiation of breastfeeding and the encouragement of mothers to breastfeed by the doctors and nurses in the obstetric departments. The Bureau of Public Health of Zhejiang Province was responsible for providing a team to regularly evaluate the Baby Friendly hospitals in the Province and to maintain the quality of the BFHI. In this atmosphere of encouragement, breastfeeding initiation rates improved. Almost all mothers understood the benefits of breastfeeding from antenatal classes, from postnatal education or from the media.

In our study more than 96% of mothers were breastfeeding their infants at discharge from hospital. The initiation of breastfeeding has returned to higher levels in the past two decades, and the rates of breastfeeding initiation in Zhejiang are now higher than reported from other provinces in China. A study of infant feeding in Xinjiang Uygur Autonomous China found an 'any breastfeeding' rate of 92.2% at discharge [6]. The 'Beijing and Four Provinces Study' found that the 'ever breastfed' rate was 90.1% in China in 2002 [10]. This rate was similar to an Australia infant feeding study from West Australia where 93.5% of mothers were breastfeeding at discharge from hospital [11].

The 'any breastfeeding' rate for the city is higher than the rate reported for Shanghai, the closest large city to Hangzhou in the 1980s [2]. This probably reflects a renewed interest in breastfeeding in China. However the situation for 'exclusive breastfeeding' is not as encouraging. The WHO reported the 0–6 months exclusive breastfeeding rate in China to be 51% [12]. Our results for 'exclusive breastfeeding' are far below this figure and may reflect different methodology. The WHO-UNICEF methodology relies on 24 hour recall of no foods or fluids apart from breast milk in the past 24 hours. China is a vast country with a huge population and another reason for the difference may be sample selection. The variation from our study suggests that larger and more widespread studies of breastfeeding are needed and they should preferably use the longitudinal methodology used in this study. Another factor influencing breastfeeding in the city is the economic pressure of the developing economy. In an ethnographic study of women in Beijing, Gottschang summarized the pressures on urban mothers: "global intervention in the form of the WHO-UNICEF sponsored Baby Friendly Hospital Initiative promotes breastfeeding as a women's duty at the same time that market forces counter this message" [13] (p. 64).

Most mothers had an understanding of the need for breastfeeding at the beginning of their infant's life, but the exclusive breastfeeding rates after discharge from hospital declined rapidly. The exclusive breastfeeding rate was considerably lower than the national target of 80% of babies being exclusively breastfed until four months of age. In our study the exclusive breastfeeding rate at discharge was only one third (38.0%) in the city and even in the suburban and the rural areas, the rates were 63.4% and 61.0% respectively. Prelacteal feeds were common in the initial days after birth, details of which have previously been reported [9]. The exclusive breastfeeding rate in Zhejiang was lower than that in Xinjiang Uygur Autonomous Region, China and in Vietnam, where the rates at discharge were 66.2% [6] and 83.6% respectively [7]. However the exclusive breastfeeding rate at six months in Xinjiang was similar to the rural area in this study.

At three months, one-half of the mothers were regularly giving their infants some infant formula. A common reason for giving formula or other complementary food is the belief that 'the more or the quicker the baby gained weight, the healthier the baby is'. In the Chinese culture, parents and grandparents are devoted to their children and with the 'one child policy' the centrality of the child in Chinese culture has increased in recent decades. Thus there is always cultural pressure to give the infant supplementary foods.

The breastfeeding rates found in this study are well below international and national targets. If the rates found in this study reflect national trends in China, it would mean that China is falling behind in its quest to meet the Millennium Goals. This suggests that further health promotion programs for breastfeeding and particularly for the extension of the period of exclusive breastfeeding are required. There needs to be continuing monitoring of the implementation of the Baby Friendly Hospital principles in the Province. Further research is needed into ways of increasing community support for breastfeeding and increasing the number of 'Baby Friendly' workplaces.

There are some limitations that need to be considered when interpreting the results of this study. The hospitals used were selected to be representative of their locations, but a larger probability sample would be required to be certain that the selected sample represented Zhejiang Province. A series of focus group discussions and/or in-depth interviews with mothers and the extended families would help in understanding further details about breastfeeding problems and beliefs about infant growth and supplementary feeds.

## Conclusion

This is the first longitudinal cohort study published on infant feeding practices in city, suburban and rural areas in Zhejiang province, an economically advanced area typical of eastern China. The overall 'any breastfeeding' rate was high before discharge at 96.5% in city, 96.8% in suburban and 97.4% in rural areas. The 'exclusive breastfeeding' rates in city, suburban and rural areas before discharge were 38.0%, 63.4% and 61.0%. The exclusive breastfeeding rate was lower than the national target at discharge and also during the whole follow-up period until the infant was six months of age. The lower exclusive breastfeeding trend was most marked in the city. More studies are needed to find the detailed reasons related to the low rates of exclusive breastfeeding.

## Competing interests

The authors declare that they have no competing interests.

## Authors' contributions

**Figure 4:**
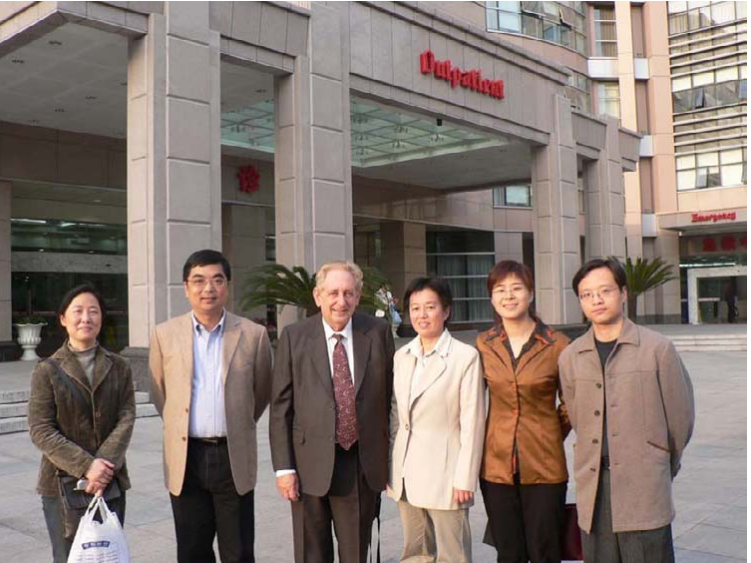
Authors (XX 2nd from left, CWB and LQ) and Hospital Staff, Women's Hospital, School of Medicine, Zhejiang University, PR China.

All authors contributed to the study (see Figure 4). LQ designed the research, collected and analyzed data, drafted the manuscript. YZ analyzed data and revised the manuscript. CWB designed the research, drafted and revised the manuscript. AL analyzed data and revised the manuscript. XX designed the research, collected data and revised the manuscript.
